# Age-Dependent Effects of Catechol-*O*-Methyltransferase (*COMT*) Gene Val^158^Met Polymorphism on Language Function in Developing Children

**DOI:** 10.1093/cercor/bhw371

**Published:** 2016-11-30

**Authors:** Lisa Sugiura, Tomoko Toyota, Hiroko Matsuba-Kurita, Yoshimi Iwayama, Reiko Mazuka, Takeo Yoshikawa, Hiroko Hagiwara

**Affiliations:** 1 Department of Language Sciences, Graduate School of Humanities, Tokyo Metropolitan University, Hachioji, Tokyo 192-0397, Japan; 2 Research Institute of Science and Technology for Society (RISTEX), Japan Science and Technology Agency (JST), Chiyoda-ku, Tokyo 100-0004, Japan; 3 Research Center for Language, Brain and Genetics, Tokyo Metropolitan University, Hachioji, Tokyo 192-037, Japan; 4 Laboratory for Molecular Psychiatry, RIKEN Brain Science Institute, Wako, Saitama 351-0198, Japan; 5 Laboratory for Language Development, RIKEN Brain Science Institute, Wako, Saitama 351-0198, Japan

**Keywords:** catecholamine, catechol-*O*-methyltransferase (COMT), children, default mode network (DMN), development, dopamine, functional near-infrared spectroscopy (fNIRS), genotype, language, preadolescence

## Abstract

The genetic basis controlling language development remains elusive. Previous studies of the catechol-*O*-methyltransferase (COMT) Val^158^Met genotype and cognition have focused on prefrontally guided executive functions involving dopamine. However, *COMT* may further influence posterior cortical regions implicated in language perception. We investigated whether *COMT* influences language ability and cortical language processing involving the posterior language regions in 246 children aged 6–10 years. We assessed language ability using a language test and cortical responses recorded during language processing using a word repetition task and functional near-infrared spectroscopy. The *COMT* genotype had significant effects on language performance and processing. Importantly, Met carriers outperformed Val homozygotes in language ability during the early elementary school years (6–8 years), whereas Val homozygotes exhibited significant language development during the later elementary school years. Both genotype groups exhibited equal language performance at approximately 10 years of age. Val homozygotes exhibited significantly less cortical activation compared with Met carriers during word processing, particularly at older ages. These findings regarding dopamine transmission efficacy may be explained by a hypothetical inverted U-shaped curve. Our findings indicate that the effects of the *COMT* genotype on language ability and cortical language processing may change in a narrow age window of 6–10 years.

## Introduction

Language is one of the higher cognitive functions unique to humans. It consists of phonology, syntax, and semantics, and these independent components interact and together promote comprehension and utterances. Language is formed by interactions between genes and experience; however, the genetic basis and signaling pathways controlling language development remain largely elusive.

Here, we focus on the gene for catechol-*O*-methyltransferase (COMT), which has been widely investigated regarding its involvement in cognitive function and psychiatric illnesses. Evidence has implied a dopaminergic influence on language ability (Barnett et al. 2007a; Prata et al. 2009; Gaysina et al. 2013); however, dopaminergic influences on language ability, perception, or processing have not been actively explored, which motivated us to investigate their impact on language functions. The human *COMT* gene on chromosome 22q11 has a functional Val^158^Met polymorphism, in which the Val allele exhibits enhanced enzymatic activity relative to the Met allele (Lotta et al. 1995; Lachman et al. 1996). Thus, the high-activity Val allele results in faster inactivation of extracellular dopamine in the brain, particularly the prefrontal cortex (PFC) (Chen et al. 2004). Therefore, Val^158^Met influences the efficiency of prefrontally guided cognitive function, specifically executive functioning, working memory, fluid intelligence, and attentional control (Egan et al. 2001; Barnett et al. 2007a, 2007b; Flint and Munafo 2007; Barnett et al. 2008, see review for Witte and Flöel 2012). Previous studies have extensively investigated prefrontally mediated cognitive functions (Mier et al. 2010). Moreover, an association between the *COMT* genotype and cognitive performance has also been identified in the peri-Sylvian cortex during a verbal fluency task (Prata et al. 2009) and the parietal region during an arithmetic working memory task (Tan et al. 2007) and a visuospatial working memory task (Dumontheil et al. 2011). Therefore, catecholamines may affect the activity of multiple cortical regions depending on the cognitive domains.

Egan et al. (2001) reported that the *COMT*^158^Val allele was associated with reduced performance on the Wisconsin Card Sorting Test and increased task-related prefrontal activation assessed via functional magnetic resonance imaging (fMRI). Several studies using cognitive tasks assessing memory and executive functions have subsequently indicated better performance in Met carriers compared with Val carriers (Mattay et al. 2003; Bishop et al. 2006; Caldu et al. 2007; Bertolino et al. 2008; Enoch et al. 2009). In these studies, a better performance was associated with lower brain activation in the PFC. Increased brain activation in Val carriers was interpreted as less efficient processing because of lower dopamine transmission efficacy (Bertolino et al. 2006, 2008; see review for Witte and Flöel 2012); however, not all studies are consistent (Barnett et al. 2008; Prata et al. 2009).

Recent studies have suggested that the effects of the *COMT* genotype on cognitive function may vary over a specific age range, possibly as a result of age-related changes in the brain dopamine system (Witte and Flöel 2012). Previous findings support the hypothesis that optimal cognitive function is associated with optimal brain dopamine signaling efficacy, thereby suggesting an inverted U-shaped response curve (Goldman-Rakic et al. 2000; Lindenberger et al. 2008; Nagel et al. 2008). Advancing age is assumed to shift individuals toward the left-hand side of the curve that relates dopamine signaling to cognitive performance based on an age-related decline in dopamine signaling efficacy (Volkow et al. 1998; Erixon-Lindroth et al. 2005; Floel et al. 2005). The deleterious effects of this leftward shift should be particularly pronounced in individuals with relatively low dopamine signaling efficacy in young Val carriers. By contrast, Mattay et al. (2003) suggested that excess dopamine shifts individuals toward the right-hand side of the curve where the Met allele is associated with impaired cognitive function.

Despite the abundance of studies that have investigated the effects of *COMT* on prefrontally guided functions in adults, similar studies of children and adolescents are sparse. Behavioral studies have indicated an association between working memory performance and the *COMT* polymorphism in children and adolescents (Diamond et al. 2004; Wahlstrom et al. 2007; Barnett et al. 2009). Of the few relevant neuroimaging studies, Dumontheil et al. (2011) investigated PFC functioning and working memory performance in a normal population with an age range of 6–20 years. They identified *COMT* genotype effects (superior performance for individuals with the Met allele compared with the Val allele) after the age of 10 years. These studies focused on prefrontally guided working memory functions. The effects of the *COMT* polymorphism on posterior and prefrontal cognitive functions other than working memory during development (before brain system maturity) are largely unknown.

Another question is whether the effects of the *COMT* genotype are static or variable during development. In the previously discussed study by Dumontheil et al. (2011), an age-dependent *COMT* effect was highlighted for both behavioral performance and cortical activation. They reported that the Val allele tended to be associated with superior performance on a visuospatial working memory task at younger ages (6–10 years) and the Met allele was beneficial after the age of 10. In the study by Barnett et al. (2007a), the genotype significantly affected executive function and verbal IQ, and subsequent analyses that included sex as a factor indicated that significant genotype effects were identified in boys and, importantly, were significantly greater in pubertal compared with prepubertal boys. Furthermore, another relevant study of the *COMT* gene in children (Gaysina et al. 2013) assessed verbal and non-verbal cognition at ages 8 and 15 years using a longitudinal design. In this study, *COMT* rs737865 was associated with reading comprehension, verbal ability, and global cognition at age 15 years in pubescent boys, but not at age 8; however, these differences were not significant following multiple comparison analyses.

Given the previous findings, we aimed to investigate the role of the Val^158^Met *COMT* polymorphism as an underlying genetic mechanism in the development of language function. More specifically, the present study examined whether the *COMT* polymorphism affected language functions in children 6–10 years of age (preadolescence) and whether a potential effect was age-dependent. We measured cortical hemodynamic changes in a sample of 246 normally developing elementary school-aged children using functional near-infrared spectroscopy (fNIRS) while the children performed a word repetition task in their first language (Japanese). Notably, previous studies have reported that dopaminergic neurotransmission dynamically changes during the preadolescent and adolescent years and increases to peak levels during this period (Kalsbeek et al. 1988; Rosenberg and Lewis 1994; Koga et al. 2016).

## Materials and Methods

### Participants

Participants were 246 healthy Japanese elementary school children (123 boys and 123 girls, aged between 6 and 10 years, with a mean age of 8.92 years, standard deviation [SD] = 0.80) who satisfied the inclusion criteria. As the details of the inclusion criteria were reported previously (Sugiura et al. 2011), a brief description is given here. Nonnationals and participants with psychiatric disorders were excluded from the analyses. The Edinburgh Handedness Inventory (Oldfield 1971) was used to determine hand dominance, and only right-handed participants were used for this study. Each participant's parent provided written, informed consent before entering this study. All of the procedures in this study were approved by the Ethics Committees of Tokyo Metropolitan University and RIKEN and were conducted according to the principles expressed in the Word Medical Association Declaration of Helsinki (2008).

### DNA Extraction and *COMT* Genotyping

Saliva for DNA extraction was obtained from all subjects using an Oragene DNA Self-Collection Kit (tube format OG-300, disc format OG-250, DNA Genotek). DNA was extracted from saliva samples using an Agencourt DNAdvance Kit (Beckman Coulter). All genotyping was performed in a manner that was blind to the phenotype measures. We used Custom TaqMan SNP Genotyping Assays products (Life Technologies) to score the *COMT* Val^158^Met genotypes based on the TaqMan assay method (Life Technologies). Genotypes were determined using an ABI7900 sequence detection system instrument (Life Technologies) and the SDS v2.4 software package (Life Technologies).

### Experimental Task, fNIRS Data Acquisition and Analyses

We utilized fNIRS (ETG-4000, Hitachi Medical Co.) to monitor cortical hemodynamic changes. The machine was installed in a mobile laboratory set in a truck and transported to elementary schools, as described in our previous study (Sugiura et al. 2011). Briefly, a 3 × 5 array of optodes containing 8 laser diodes and 7 light detectors, which were alternately placed at an inter-optode distance of 3 cm (typical distance used for all ages from infants to adults: Rossi et al. 2012) and yielded 22 channels, was applied on each side of a participant's head. Optical data from the individual channels were collected at 2 different wavelengths (695 and 830 nm) and analyzed using the modified Beer–Lambert Law for a highly scattering medium (Cope et al. 1988). Changes in oxygenated [oxy-Hb], deoxygenated [deoxy-Hb], and total hemoglobin [total-Hb] signals were calculated in units of millimolar·millimeter (mmol·mm) (Maki et al. 1995).

We used an aural repetition task with high- and low-frequency word conditions provided in the children's native language during hemodynamic measurements because words are the basic building blocks of language that underlie higher level linguistic processing, including syntax and discourse. Repetition is thought to reflect linguistic capability (Gathercole and Baddeley 1989, 1990), and many studies in various research domains have demonstrated that repetition facilitates grammatical and lexical development (Corrigan 1980; Snow 1981, 1983; Kuczaj 1982; Speidel and Nelson 1989; Perez-Pereira 1994). We used 60 words that included 30 high-frequency words and 30 low-frequency words. High-frequency words are defined as having more than 50 occurrences per 1 million words, whereas the low-frequency words have less than 5 occurrences per 1 million. All words were taken from a corpus (Amano and Kondo 2000). Children were seated and instructed to overtly repeat the words as heard from a speaker. Each word was repeated immediately after being heard. Within each condition, the stimuli were presented in blocks of 5 words. A total of 6 blocks were presented for each condition, with each block having a duration of 35 s. Each block contained a 5-s prestimulus period, a 15-s stimulus period, and a 10-s recovery period, followed by a 5-s poststimulus period. The hemodynamic response is gradually restored to baseline after the stimulus period and returns to near baseline levels after several seconds; thus, a recovery period of 10 s was set for the complete recovery of the hemodynamic response before the poststimulus period. The fNIRS data were preprocessed using the Platform for Optical Topography Analysis Tools (POTATo) (Adv. Res. Lab., Hitachi Ltd.) as reported previously (Sugiura et al. 2011). For each individual set of hemoglobin data, we extracted data blocks from the time course data. For each channel, a baseline correction was performed by linear fitting according to the mean value of the 5-s prestimulus and 5-s poststimulus. For each child, the mean changes in [oxy-Hb] and [deoxy-Hb] signals during the stimulus and recovery periods were calculated, and the activity recorded during the stimulus and recovery periods was compared with the activity recorded during the baseline periods using Student's *t*-tests (2-tailed, *P* < 0.05, Bonferroni corrected for family-wise errors) for each independent channel (22 channels in each hemisphere) for each of the 2 conditions (the statistical results of the independent channels used in the present study are shown in Supplementary Table 1). All blocks that were affected by movement artifacts were removed, and participant data that contained a minimum of 3 of 6 data blocks for each task were used. We utilized the channels that had greater than a 60% survival rate of data across all subjects after controlling for movement. As channels 15 and 20 did not reach the criterion due to movement in the temporal muscles for both conditions, they were not used for further analyses. We present the results of the [oxy-Hb] analyses because the results of the [oxy-Hb] and [deoxy-Hb] analyses were consistent with an increased sensitivity in detecting significance in the [oxy-Hb] signals. We employed a virtual registration method (Tsuzuki et al. 2007) to register fNIRS data to the Montreal Neurological Institute (MNI) standard brain space.

Before testing the genotype effects, the language-related regions of interest (ROIs) were defined based on the results of the spatial registration and by referring to a standard macroanatomical atlas (Automatic Anatomical Label) (Tzourio-Mazoyer et al. 2002) in the channels that exhibited a statistically significant increase in [oxy-Hb] for at least one combination of *COMT* genotype, word-frequency condition, and cerebral hemisphere. As our previous analyses revealed that some adjacent cortical subregions exhibited similar characteristics of activation patterns and given the limited information available regarding catecholamine modulation or *COMT* effects in cortical subregions, we simply defined 4 ROIs bilaterally to examine whether *COMT* genotypes played a role (as shown in Figure 3: 1) the temporal region: TR, the vicinity of Wernicke's area (Brodmann areas, BA41, 42 and BA21, 22); 2) the angular gyrus: AG (BA39); 3) the supramarginal gyrus: SMG (BA40); and 4) the frontal region: FR, part of Broca's area (BA44, 45)). Overall [oxy-Hb] signal levels in a single ROI were obtained by calculating the mean [oxy-Hb] signal levels of all the channels within the ROI. *P* values were false discovery rate (FDR) corrected for ROI analyses with a significance level of *P* < 0.05 after correction for multiple testing.

### Behavioral Data Acquisition and Analyses

To examine comprehensive language ability, participants were administered a language test (Japanese) that assessed lexical knowledge, reading comprehension, listening comprehension, and writing ability. This test is also used for annual nationwide surveys of academic achievement in elementary school children who are conducted by the Ministry of Education, Culture, Sports, Technology, and Science (MEXT), Japan. Each child took a version of the test appropriate to his or her grade. The adjusted SD scores, which are widely used in Japan, were used to standardize scores from different tests.

The repetition success rates were estimated using the online behavioral data recorded during the fNIRS measurements. The veracity of the repeated words was evaluated phoneme-by-phoneme for each participant by a native Japanese speaker. Repetition success rates were calculated as described previously (Sugiura et al. 2011).

### Statistical Analyses

All statistical analyses were performed using the SPSS statistical package (SPSS Inc.). To determine whether the *COMT* genotypes affect linguistic performance (language ability), we initially analyzed the behavioral data (language test scores). Previous studies of *COMT* effects on cognition and neural activity in children and adolescents have indicated interactions of genotype and age (Barnett et al. 2007a; Dumontheil et al. 2011; Gaysina et al. 2013); thus, we considered age a potential factor in the analysis. The age range of our participants was not large; thus, we divided them into 2 age groups (young [*n* = 123, mean age ± SD: 8.3 ± 0.5; ages of 6–8 years] and old [*n* = 123, mean age ± SD: 9.6 ± 0.5; ages of 9–10 years]) to examine the presence or absence of the effect. A 2 × 2 analysis of covariance (ANCOVA) was used to identify main and interaction effects of the *COMT* genotype (Met carriers (Met/Met [MM] + Val/Met [VM]) and Val homozygotes (Val/Val [VV])) and age (young and old) on the language test scores. No sex-specific or sex-interaction effects were identified in our preliminary analysis; however, we included sex as a covariate for confirmation because several studies have suggested a sex-specific effect, with stronger influences of *COMT* in boys than in girls (Barnett et al. 2007a; Gaysina et al. 2013). When a significant interaction was detected between *COMT* genotype and age group, the simple main effects were evaluated using unpaired *t*-tests to test more specifically for differences in the interaction. *P* values of <0.05 (2-sided) were considered to be significant.

Regarding the cortical response during word processing, 4-way repeated-measures ANCOVAs were conducted for 4 ROIs, with sex as a covariate, to evaluate the effects of 2 between-subject factors: 2 *COMT* genotypes (Met carriers (MM + VM) and Val homozygotes (VV)) and 2 age groups (young and old), as well as 2 within-subject factors: 2 task conditions (high-frequency and low-frequency word conditions) and 2 hemispheres (left and right). When ANCOVA yielded a significant interaction between the *COMT* genotype and other factors, post hoc simple main effect analyses were performed using *t*-tests. *P* values were FDR corrected for multiple testing with a significance level of *P* < 0.05 after multiple comparison correction. For FDR correction, we used the following numbers for multiple testing: 4 tests (corresponding to 4 ROIs) in ANCOVA and 2 tests (corresponding to 2 ROIs) in post hoc simple main effect analyses (because only 2 ROIs showed main effects of the *COMT* genotype in initial ANCOVA and were therefore included in further post hoc analyses).

The results of the global ANCOVA incorporating all variables in a single comparison did not indicate a significant interaction between genotype and age for the fNIRS analyses. However, because the behavioral data exhibited an interaction between genotype and age, we conducted additional analyses using unpaired *t*-tests to examine whether 2 age groups (the same age groups used in the behavioral analysis) exhibited different trends. One age group exhibited significant differences in cortical activation between the 2 genotype groups, whereas there were no differences in the other group; thus, the results of the additional analyses are also reported.

## Results

### *COMT* Genotyping Results

The participants were genotyped for the *COMT* Val^158^Met polymorphism. The frequencies of the Met homozygotes (Met/Met [MM]), Val/Met heterozygotes (Val/Met [VM]), and Val homozygotes (Val/Val [VV]) in the study population were 7.7%, 44.7%, and 47.6%, respectively. The genotype distribution was consistent with the Hardy–Weinberg equilibrium (*P* = 0.323 by χ^2^ test). The Met carriers were grouped together (MM and VM) for analyses because the rarity of Met homozygotes in our Japanese cohort compared with analogous Western populations prevents sufficient observations for a meaningful analysis. Nevertheless, following an additional statistical analysis using 1-way ANOVAs, we confirmed that there were no differences in cortical activation between MM and VM in any brain regions examined, and the ultimate results and conclusions are the same as the 2-group analysis of Met carriers (MM and VM) and VV. Thus, all statistical analyses that examined *COMT* genotype effects in the present study were conducted for 2 *COMT* genotype groups (Met carriers [*n* = 129] and Val homozygotes [*n* = 117]). No differences were identified in the ratios for age or sex according to the genotypes (Table 1).

**Table 1:** Demographic variables according to genotype

	Met/Met + Val/Met	Val/Val	df	*t*	*P*
Genotype counts (%frequency)	129 (52.4%)	117 (47.6%)			
Age in years (±SD)	8.97 (0.823)	8.89 (0.775)	244	0.804	0.422
Boy/girl	63/66	59/58	244	0.248	0.804

### Behavioral Performance: Language Ability

A Japanese language test was administered to assess comprehensive language ability. A 2 × 2 ANCOVA was performed, with sex as a covariate, to test the main and interaction effects of the *COMT* genotype (Met carriers vs. Val homozygotes) and age (young [6–8 years] vs. old [9–10 years]) on the language test scores. We identified significant differences in the test scores between the *COMT* genotype groups and between the age groups (Table 2). The *COMT* genotype effect indicated a better performance by the Met carriers compared with the Val homozygotes. Regarding the age effect, the old group exhibited a better performance compared with the young group. A sex effect was not identified. Importantly, a significant interaction was identified between the *COMT* genotype and age.

**Table 2:** Results of 2 × 2 ANCOVA, with sex as a covariate, indicating the main effects and an interaction of the *COMT* genotype and age on Japanese language test scores

Source of variation	SS	df	MS	*F*	*P*	Remarks
*COMT*	152.857	1, 235	152.857	3.937	0.048*	MM + VM > VV
Age	155.763	1, 235	155.763	4.012	0.046*	young < old
*COMT* x age	174.686	1, 235	174.686	4.500	0.035*	
Sex	3.387	1, 235	3.387	0.087	0.768 (n.s.)	

Notes: Asterisks indicate significant results (*P* < 0.05). SS, sum of squares; df, degrees of freedom; MS, mean squares; and *F*, variance ratio. MM, Met/Met; VM, Val/Met; VV, Val/Val.

#### Post hoc Results: COMT Genotype and Age Effects on Language Test Scores

**Figure 1.**
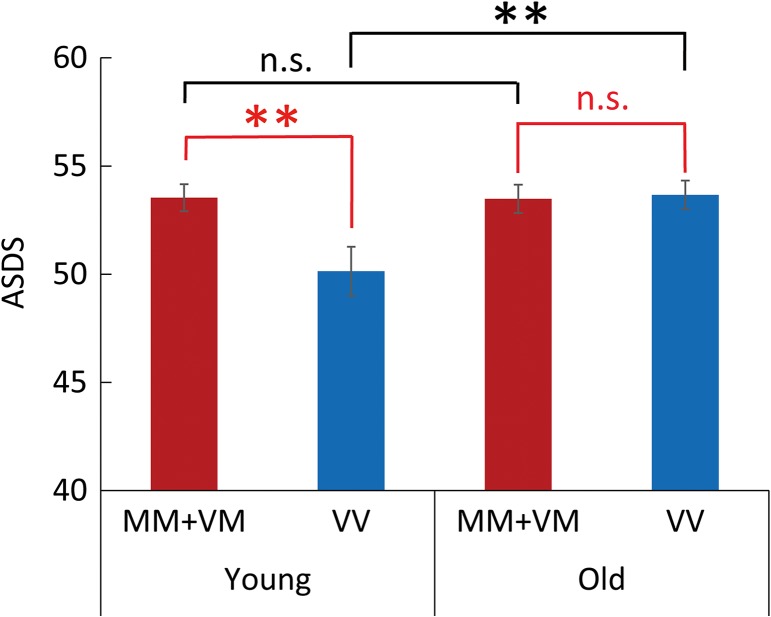
Effects of *COMT* genotype and age on language test scores. A language test was administered to assess comprehensive language ability. A significant main effect of the *COMT* genotype on the language test score was identified between the Met carriers (MM + VM) and Val homozygotes (VV) (*t*(92.5) = 2.628, *P* = 0.010**, MM + VM > VV) in the young group, whereas no main effect of the *COMT* genotype was identified in the old group. Regarding the age effect on the language test score, no main effect was identified between the young and old groups for the Met carriers (MM + VM); however, a significant main effect of age was identified between the young and old groups for the Val homozygotes (VV) (*t*(94.8) = −2.686, *P* = 0.009**, young < old). ASDS, adjusted standard deviation scores. Error bars indicate standard error (SE). Asterisks indicate significant results, and n.s. indicates not significant.

Because of the significant interaction detected between the *COMT* genotype and age, post hoc simple main effect analyses were performed using unpaired *t*-tests. The statistically significant and non-significant differences in the language test scores between the Met carriers (MM + VM) and Val homozygotes (VV) for the young and old groups, as well as those between the young and old groups for the Met carriers (MM + VM) and Val homozygotes (VV), are summarized in Figure 1. A significant main effect of the *COMT* genotype on the language test scores was identified between the Met carriers and Val homozygotes (*t* (92.5) = 2.628, *P* = 0.010**, MM + VM > VV) in the young group. By contrast, no main effect of the *COMT* genotype was identified in the old group. Additionally, while a significant age effect on the test scores was identified among the Val homozygotes (*t* (94.8) = −2.686, *P* = 0.009**, young < old), no age effect was found among the Met carriers.

### Cortical Responses During Language Processing

#### COMT Genotype Effects on Cortical Activation During Language Processing

**Figure 2.**
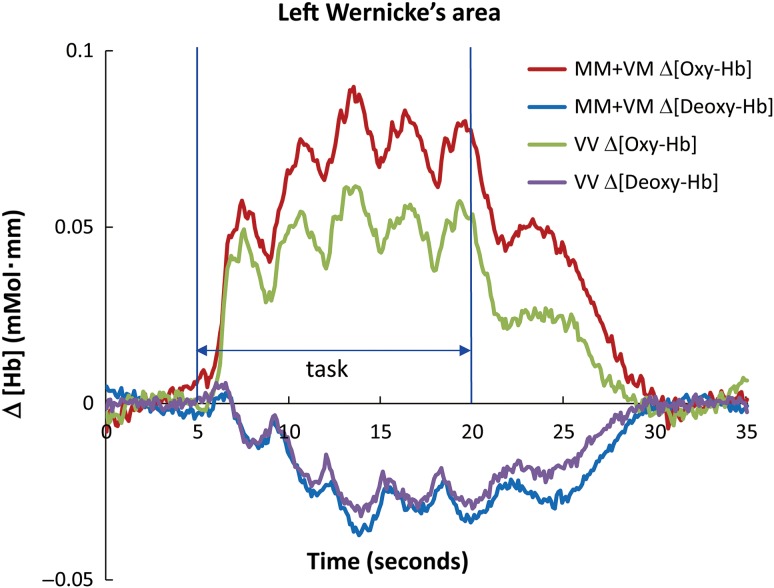
A typical time course depicting grand-averaged [oxy-Hb] and [deoxy-Hb] changes in Wernicke's area for the different *COMT* genotype carriers. The left posterior temporal region (Wernicke's area) exhibited a main effect for the *COMT* genotype during the high-frequency word condition. Red line: Δ [oxy-Hb] and blue line: Δ [deoxy-Hb] for Met carriers; green line: Δ [oxy-Hb] and purple line: Δ [deoxy-Hb] for Val homozygotes; vertical blue lines: task onset and end time points. NIRS activation studies on normal adults demonstrated that neuronal activation generally causes an increase in Oxy-Hb with a concomitant decrease in Deoxy-Hb within the activated cortical area.

Differences in cortical activation between the Met carriers and Val homozygotes during word processing were compared via ROI-based analyses (4 ROIs: TR, AG, SMG, and FR). As an example, the temporal dynamics of cortical activation are illustrated in Figure 2. Both genotype groups exhibited increases in [oxy-Hb] and decreases in [deoxy-Hb], which represents a response pattern consistent with previous studies (Sakatani et al. 1998). This typical time course of [oxy-Hb] and [deoxy-Hb] of grand-averaged data demonstrates increased cortical activation in the temporal region of Met carriers compared with Val homozygotes.

**Figure 3.**
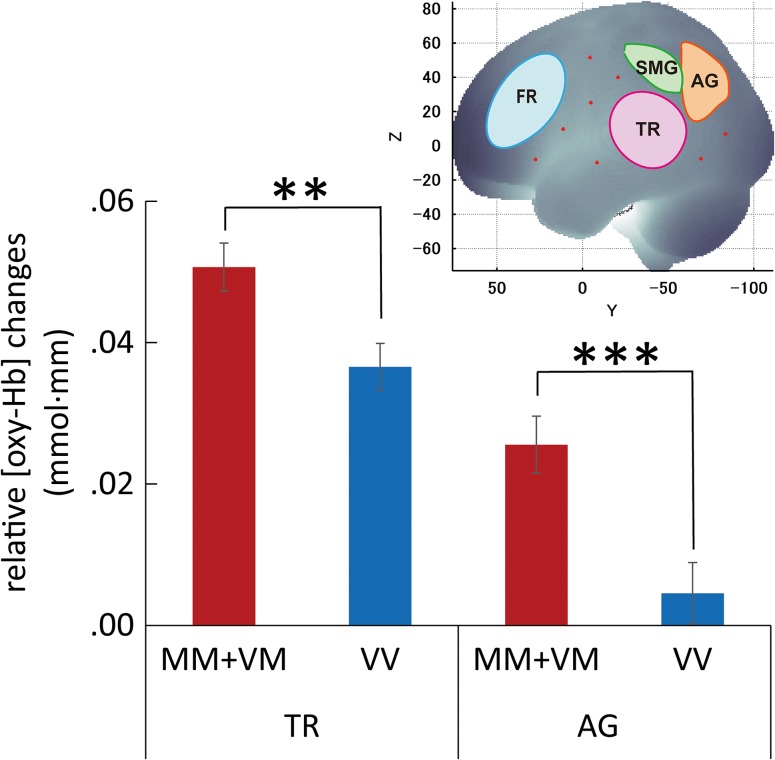
Effects of *COMT* genotype on cortical activation during word processing. A summary of the ROI-wise cortical activation during high-frequency word processing is shown. Bilateral language-related ROIs were defined on an MRI template image that represented brain anatomy in accordance with the MNI space (upper right); the bar graphs with statistics indicate the differences in the average cortical activation between the Met carriers (MM + VM) and Val homozygotes (VV) in the temporal region and angular gyrus. (The effects of the *COMT* genotype were not identified in the frontal region and supramarginal gyrus.) The vertical axes represent the relative changes in [oxy-Hb] in units of millimolar·millimeter (mmol·mm), and the error bars indicate SE. *P* values are based on FDR corrections for 2 tests with a significance level of *P* < 0.05 after correction for multiple testing. Asterisks indicate significant results (**P < 0.01, ***P < 0.001), and n.s. indicates not significant. TR,  temporal region, including Wernicke's area; AG, angular gyrus; SMG, supramarginal gyrus; and FR, frontal region, including Broca's area.

We initially conducted 4-way repeated-measures ANCOVAs for 4 ROIs, with sex as a covariate, to examine the effects of the *COMT* genotype (Met carriers (MM + VM) and Val homozygotes (VV)), age group (young and old), task condition (high-frequency and low-frequency word conditions), and hemisphere (left and right hemispheres). *P* values were FDR corrected for 4 tests (for 4 ROIs) with a significance level of *P* < 0.05 after multiple testing correction. Because of space limitations, the complete results are presented in Supplementary Table 2, and only the results of the *COMT* genotype effects in 4 ROIs are listed in Table 3. Significant main effects of the *COMT* genotype were identified in the angular gyrus and temporal region, including Wernicke's area. There were no significant main or interaction effects of the *COMT* genotype in the supramarginal gyrus or frontal region, including Broca's area. Therefore, these 2 ROIs were not included in further analyses. In addition to the main effects of the *COMT* genotype, an interaction between the *COMT* genotype and the task condition was identified in the angular gyrus (*P* < 0.05, details in Supplementary Table 2). Because of this interaction and the finding that the differences in cortical responses between task conditions in the temporal region were not small (although they were not significant), post hoc analyses were performed for 2 separate task conditions for the 2 ROIs in which the genotype effects were identified. The factors age, hemisphere, and sex, which had no significant interactions with the *COMT* genotype, were not included in the post hoc analyses. Hereafter, *P* values were FDR corrected for 2 tests (for 2 ROIs) with a significance level of *P* < 0.05 after multiple testing correction. As summarized in Figure 3, the unpaired *t*-test results indicated significant differences in the cortical activation between the Met carriers (MM + VM) and Val homozygotes (VV) in the angular gyrus (*t*(244) = 3.552, uncorrected *P* < 0.001***, FDR-corrected *P* < 0.001***, MM + VM > VV) and the temporal region, including Wernicke's area (*t*(244) = 2.947, uncorrected, *P* = 0.0035**, FDR-corrected *P* = 0.0035**, MM + VM > VV) for the high-frequency word condition. By contrast, no significant *COMT* genotype effects were identified in the angular gyrus or temporal region for the low-frequency word condition. These findings indicate that the *COMT* genotype effects are more pronounced when familiar words are processed than when unfamiliar words are processed.

**Table 3:** Effects of *COMT* genotype on cortical activation during word processing

Brain area	SS	df	MS	*F*	*P* uncorrected	*P* corrected	Remarks
TR	0.028	1237	0.028	7.234	0.008	0.031*	MM + VM > VV
AG	0.041	1240	6.751	6.751	0.010	0.020*	MM + VM > VV
SMG	0.006	1236	0.921	0.921	0.338	n.s.	
FR	0.000	1239	0.004	0.004	0.952	n.s.	

Notes: Statistical analyses using 4-way repeated-measures ANCOVAs were conducted for 4 ROIs, with sex as a covariate, to assess the effects of the *COMT* genotype (Met carriers (MM + VM) and Val homozygotes (VV)), age group (young and old), task condition (high-frequency and low-frequency word conditions), and hemisphere (left and right hemispheres). As a result of space limitations, the complete results are presented in Supplementary Table 2, and only the results of the *COMT* genotype effects are listed here. *P* values are based on FDR corrections for 4 tests (for 4 ROIs) with a significance level of *P* < 0.05 after multiple testing correction. Thus, the smallest *P* value is compared with 0.05/4 = 0.0125, the second smallest *P* value is compared with 0.05 × 2/4 = 0.025, the third smallest *P* value is compared with 0.05 × 3/4 = 0.0375, and the fourth smallest *P* value is compared with 0.05 × 4/4 = 0.05. Asterisks indicate significant results (*P < 0.05), and n.s. indicates not significant. SS, sum of squares; df, degrees of freedom; MS, mean squares; *F*, variance ratio; TR, temporal region, including Wernicke's area; AG, angular gyrus; SMG, supramarginal gyrus; and FR, frontal region, including Broca's area.

#### Additional Analyses: Difference in the COMT Genotype Effect Between the 2 Age Groups

The results of the global ANCOVA incorporating all variables in a single comparison did not indicate a significant interaction between genotype and age for the fNIRS analyses. Nevertheless, because the language performance exhibited an interaction between genotype and age, additional fNIRS analyses were conducted to determine whether 2 age groups (the same age groups used in the behavioral analysis) exhibited different trends. Specifically, for the 2 separate age groups (young and old), we examined the effects of the *COMT* genotype on cortical activation in the 2 ROIs for the high-frequency word condition, in which significant *COMT* genotype effects were identified. Statistical analyses using unpaired *t*-tests indicated significant effects of the *COMT* genotype on cortical activation in 2 ROIs (*t*(121) = 3.614, uncorrected *P* < 0.001***, FDR-corrected *P* < 0.001***, MM + VM > VV for AG; *t*(121) = 2.905, uncorrected *P* = 0.0044**, FDR-corrected *P* = 0.0044**, MM + VM > VV for TR) in the old group but not in the young group. In addition, the age effects for the 2 genotype groups using unpaired *t*-tests were assessed. The results indicated trend effects (*t*(115) = 1.838, uncorrected *P* < 0.069(*), FDR-corrected *P* < 0.069(*), young > old for AG; *t*(115) = 2.006, uncorrected *P* = 0.047*, FDR-corrected *P* = 0.094(*), young > old for TR) for the Val homozygotes (VV); however, for the Met carriers (MM + VM), no significant differences in cortical activation were identified between the 2 age groups for either of the 2 ROIs.

The omnibus ANCOVA did not identify significant interactions between the *COMT* genotype and age for cortical responses; thus, the age-dependent genotype effects on cortical responses may not be as significant as the effects on language performance. However, there appears to be a critical difference in the genotype effects between the 2 age groups.

The repetition success rates between the 2 genotype groups were also compared using unpaired *t*-tests for both age groups. Regarding the high-frequency word condition, both age groups exhibited ceiling effects because they obtained nearly perfect success rates in this condition (Supplementary Table 3). A survey on the word list used for the task indicated that the mean semantic knowledge was significantly greater for the high-frequency words compared with the low-frequency words (Supplementary Fig. 1). Regarding the low-frequency word condition, there were no significant differences in the success rates between the 2 genotypes for both age groups (Supplementary Table 3), which is consistent with the fNIRS data.

## Discussion

In the present study, we examined 246 elementary school-aged children to determine the effects of the *COMT* Val^158^Met polymorphism on language performance and fNIRS-based cortical responses during language processing. The results demonstrated significant differences in language ability and cortical responses in the posterior language areas between 2 *COMT* genotype groups (Met carriers vs. Val homozygotes). Importantly, 1) age-dependent effects were identified, and 2) *COMT* genotype effects were not observed in the prefrontal region; however, they were observed in posterior cortical regions. We discuss these 2 findings in light of previous studies.

### Age-Dependent *COMT* Genotype Effects on Language Function

Met carriers exhibited better performance compared with Val homozygotes on the language test in the young group. By contrast, the 2 genotype groups exhibited equal performances in the old group. These findings indicate slower language development in Val homozygotes compared with Met carriers. Regarding the cortical responses, during high-frequency word processing, both genotype groups exhibited equal activation in the young group, whereas Val homozygotes exhibited significantly less activation compared with Met carriers in the old group. Based on the results of language ability and cortical responses, the present study suggests that the *COMT* Val^158^Met polymorphism affects cortical language processing and language ability in children younger than 10 years of age.

Regarding age effects, Met carriers did not exhibit significant differences between the young and old groups in language ability or cortical responses. However, the Val homozygotes exhibited significantly better language performance and decreased cortical activation with age. These findings suggest that Met carriers attain more advanced language development compared with Val homozygotes in terms of language ability during the early elementary school years (ca. 6–8 years), whereas Val homozygotes exhibit significant language development during the later elementary school years. Consequently, children with both genotypes exhibit equal language performance at approximately 10 years of age. Our findings on children suggest that the *COMT* genotype affects cortical language processing and language ability; however, its effects are variable during a specific window of development.

### Potential Mechanisms for Age-Dependent *COMT* Genotype Effects on Language Function

The outperformance of the Met carriers compared with the Val homozygotes in the language test during the early elementary school years is consistent with previous studies that reported a benefit in Met carriers relative to Val homozygotes despite differences in the cognitive functions and brain regions investigated (Egan et al. 2001; Goldberg et al. 2003; de Frias et al. 2004; Bruder et al. 2005; Barnett et al. 2007b; Caldu et al. 2007; Flint and Munafo 2007; Bertolino et al. 2008; Diaz-Asper et al. 2008; Enoch et al. 2009; see review for Witte and Flöel 2012). However, both genotype groups performed equally on the language test at approximately 10 years of age. Many initial studies reported benefits for Met carriers compared with Val homozygotes; however, some studies indicated no effect or the opposite effect (e.g., Tsai et al. 2003; Stefanis et al. 2004; Ho et al. 2005; Schott et al. 2006; Winterer et al. 2006; Barnett et al. 2008; Prata et al. 2009; Dennis et al. 2010). Thus, the effects of the *COMT* polymorphism on brain function and behavior in healthy individuals have been controversial and complex.

Changes in dopamine signaling efficacy in the brain during a life span may influence genotype–phenotype correlations and may explain the age-dependency of the present results. Previous neurocomputational simulations (Li and Sikström 2002) and experimental studies of animals (Vijayraghavan et al. 2007) and humans (Mattay et al. 2003) suggest that the relationship between dopamine signaling and cognitive performance follows an inverted-U pattern (Goldman-Rakic et al. 2000; see Cools and D'Esposito 2011; Li 2013 for recent reviews). This “inverted-U relationship” indicates that there is an optimal level of dopamine transmission for the highest level of performance for a specific task, and too much or too little enzymatic activity has a negative effect on performance according to an assumed curve, as previously described. Importantly, the position of the curve along the *x*-axis (efficacy of dopamine signaling) would shift with age and genotype. Tunbridge et al. (2007) examined COMT enzyme activity in the PFC during human postnatal development. They reported a significant increase in COMT enzyme activity from neonates to adulthood in both Val^158^Met genotype groups, which may explain previous findings of protracted postnatal changes in the PFC dopamine system, particularly the age-related decrease in dopamine signaling efficacy, which accounts for the decline in cognitive performance (Volkow et al. 1998; Erixon-Lindroth et al. 2005; Floel et al. 2005).

Furthermore, the peak of the inverted U-shaped curve should be task dependent. Cools and Robbins (2004) have suggested that a single inverted U-shaped curve is insufficient for predicting performance. The effects of the *COMT* genotype on prefrontal functions exhibited interactions with age in developing children (Wahlstrom et al. 2007; Dumontheil et al. 2011). Diamond et al. (2004) identified an advantage of Met homozygotes compared with Val homozygotes in the dots-mixed task in younger children (*n* = 39, 6–14 years, mean age = 9 years). Moreover, Wahlstrom et al. (2007) determined that the Val-Met genotype was optimal in PFC-mediated cognitive tasks and performed better than both homozygote groups in older children and adolescents (*n* = 70, 9–17 years, mean age = 13). In this case, the Val-Met genotype is thought to be located around the peak of an inverted U-shaped curve corresponding to the relevant tasks and ages.

**Figure 4.**
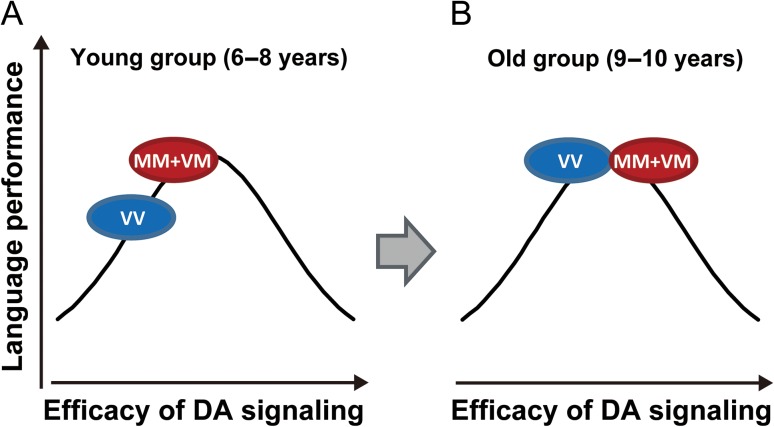
Putative inverted U-shaped relationships between language performance and efficacy of dopamine (DA) signaling in healthy children aged 6–10 years. An arbitrary Gaussian curve was used. Too much or too little cortical dopamine availability was associated with a poorer performance. (*A*) Met carriers outperformed Val homozygotes in the language test during the early elementary school years (ca. 6–8 years), which suggests Met carriers predict optimal functioning and lie closer to the apex of this curve compared with Val homozygotes. (*B*) The relative place of each allele on the inverted U-shaped curve would shift depending on individual age, and Val homozygotes exhibited significant growth in language development during the later elementary school years. Language development was exclusively demonstrated in the Val homozygotes, but not the Met carriers, and both groups performed equally on the language test in the later elementary school years (ca. 9–10 years), which suggests both groups lie closer to the apex of the inverted U-shaped curve.

The dopaminergic system is modulated by various neurochemical changes (Meyer et al. 2014) and undergoes substantial reorganization during postnatal development. D1 and D2 receptor levels in the striatum of developing children are significantly higher than those in adults in both humans (Seeman et al. 1987) and rats (Gelbard et al. 1989; Teicher et al. 1995). The peak in D1 and D2 receptor binding during adolescence and the decline toward adulthood are considerably more pronounced in the striatum than in the nucleus accumbens (Teicher et al. 1995). Adult D3 receptor density is much greater than that in developing children in the striatal regions and accumbens (Stanwood et al. 1997). Regarding the PFC, mRNA levels of D1, D2, and D4 receptors are the most abundant among all dopamine receptor transcripts; however, individual mRNA levels may change with age (Meador-Woodruff et al. 1996). In human postmortem studies of the PFC, Weickert et al. (2007) demonstrated that D1 receptor density is highest in adolescents and young adults compared with neonates, infants, adults, and aged adults. D2 receptor density is highest in neonates, and the most robust change (decrease) occurred in the aged group. D2 receptor density also appeared to increase in the adolescent group; however, this difference did not reach statistical significance. In contrast to the age-specific changes in D1 and D2 receptors, significant age-related changes in D4 receptor density were not identified. These studies suggest that the peak of the inverted U-shaped curve also depends on the brain region and the receptor type that are activated. Jucaite et al. (2010) identified a decrease in D1 receptor binding in the PFC and notably in the parietal cortex over an age span of 10–30 years. These changes in dopaminergic modulation would result in a peak in prefrontal and potentially parietal dopaminergic neurotransmission in preadolescence or early adolescence (Teicher et al. 1993; Rosenberg and Lewis 1994, 1995; Andersen et al. 1997; Wahlstrom et al. 2010). If the inverted-U relationship for the posterior cortical regions is similar to that for the PFC, the present results are consistent with previous studies, and it can be deduced that compared with Val homozygotes, Met carriers have better language ability at earlier ages as a result of increased dopamine signaling efficacy in the posterior cortical regions (Fig. 4*A*). However, at an older age, both Met carriers and Val homozygotes are located around the peak of the inverted U-shaped curve (Fig. 4*B*). This operational switch may be partly explained by the increase in D1 receptor density during the preadolescent period (Seeman et al. 1987; Teicher et al. 1995; Weickert et al. 2007).

### *COMT* Genotype Effects in Posterior Language Regions

The posterior language regions, including the posterior temporal and inferior parietal regions, play critical roles in language perception, and we identified *COMT* genotype effects in these regions.

Previous studies that have investigated *COMT* genotype effects intensively focused on prefrontally mediated cognitive functions (Barnett et al. 2007b; Flint and Munafo 2007; Barnett et al. 2008; see review for Witte and Flöel 2012); however, the *COMT* genotype is also associated with brain activation in subcortical areas during affective processing (Smolka et al. 2005; Drabant et al. 2006). Furthermore, in several working memory studies, *COMT* genotype effects have been identified in both prefrontal and parietal regions (Tan et al. 2007; Dumontheil et al. 2011).

Biological evidence suggests the possibility that the *COMT* gene influences cortical functions outside the PFC. Previous studies have indicated that the correlation between dopamine D1 receptor availability and the *COMT* Val^158^Met polymorphism is not limited to the PFC; rather, it occurs throughout the cortex (Slifstein et al. 2008). Furthermore, the COMT protein and its enzymatic activity are widely detected in the mammalian brain (Lundstrom et al. 1995; Mannisto and Kaakkola 1999). Other studies have indicated that the *COMT* gene is expressed throughout the brain, with increased levels in the frontal and temporal cortices compared with subcortical regions (Hong et al. 1998; Matsumoto et al. 2003). In an adult study, *COMT* genotype effects were also identified in the peri-Sylvian cortex, including temporal regions, in a verbal fluency task (Prata et al. 2009).

With respect to the parietal region, the training of working memory, which improves working memory capacity, is associated with changes in the density of D1 receptors in both prefrontal and parietal cortical regions (McNab et al. 2009). In addition, compared with Met homozygotes, Val homozygotes exhibited enhanced activity in the posterior parietal regions during an arithmetic working memory paradigm (Tan et al. 2007), as well as the right intraparietal sulcus and angular gyrus during a visuospatial working memory task (Dumontheil et al. 2011). Notably, single-word repetition in the native language requires limited working memory compared with the working memory paradigm. Processing familiar words requires a smaller working memory load compared with processing unfamiliar words. Therefore, the *COMT* effects identified in the posterior language regions during high-frequency word processing, but not during low-frequency word processing, in the present study would not be related to working memory functions.

### Potential *COMT* Mechanisms in Brain Regions Outside the PFC

Previous studies have identified *COMT* genotype effects simultaneously in both prefrontal and parietal regions (Tan et al. 2007; Dumontheil et al. 2011); however, the present study identified these effects in the posterior cortical regions (including the parietal region) but not in the PFC even though our task activated both regions simultaneously. This finding suggests that the effects of *COMT* on prefrontal and posterior activation may occur independently.

One potential reason why *COMT* genotype effects were not identified in the PFC may be because of the task used in the present study. Specific engagement of the temporal and parietal regions is related to the function of speech perception, whereas the frontal region is strongly related to the function of speech production. Our task required speech perception rather than speech production. Furthermore, the prefrontal activation elicited by word repetition may not be affected by changes in the activity of the dopamine system or other catecholamine systems.

The study by Stokes et al. (2011) is useful for a fundamental reconsideration of the *COMT* genotype effect on PFC activity. This study investigated whether the *COMT* genotype influenced cortical activation, particularly PFC activation, in adults using 3 fMRI tasks that are associated with the dopaminergic system. Intriguingly, they reported no significant relationships between the *COMT* genotypes and PFC activation for the 3 tasks. However, for 2 tasks, they identified *COMT* genotype effects in the posterior cingulate cortex, where deactivation was demonstrated. Their findings suggest that the *COMT* Val^158^Met polymorphism did not have direct effects on PFC activation and potentially affected the default mode network (DMN). According to the tasks, the *COMT* genotype status may indirectly impact PFC functions through the modulation of the posterior cingulate via its connections with DMN components. Recent studies have aimed to elucidate the specific role of dopamine and the *COMT* genotypes on the DMN and executive network function (Lee et al. 2011; Dang et al. 2012; Tunbridge et al. 2013). The DMN comprises a set of brain regions that exhibit highly synchronized intrinsic neuronal activation during rest and consistently decreased neural activity during goal-oriented tasks (Shulman et al. 1997). This network consists of the dorsal and ventromedial PFC, posterior cingulate cortex, precuneus, inferior parietal regions, lateral temporal cortex, and hippocampal formation (Buckner et al. 2008). The results of the present study together with the results of Stokes et al. (2011) suggest that the *COMT* genotype does not play a direct role in the modulation of PFC activation in some tasks. Rather, it modulates cognitive functions and neuronal activity in the brain regions associated with the DMN.

The other potential reason that no genotype effect was identified in the PFC may be related to the differential maturation of brain regions. Neuroimaging studies have demonstrated that anatomical growth occurs in the primary sensorimotor cortices, with the frontal and occipital poles maturing first and the remainder of the cortex developing in a parietal-to-frontal (back-to-front) direction (Gogtay et al. 2004). Studies of the DMN have demonstrated sparse connections between the parietal default regions and the PFC in early school-aged children (7–9 years old) (Fair et al. 2008). These findings indicate that the parietal region matures both structurally and functionally earlier than the prefrontal region. The PFC develops slowly until late adolescence. As the ages of our participants were 10 years and under, *COMT* effects may not have been identified in the PFC. The effects of the *COMT* genotype should be dependent on the degree of structural and functional maturation of language-related cortical regions.

### Potential Effects of the *COMT* Genotype on DMN Activity Controlling Task-Related Neuronal Responses

The directionality of the *COMT* genotype effects on brain activation (i.e., whether Val or Met carriers exhibit relatively greater cortical activation) has varied between studies depending on the task (including task demands) and brain region. With regard to the brain region, our results are consistent with the results of Prata et al. (2009) in that the Met allele was associated with increased activation in the peri-Sylvian cortex compared with that of the Val allele in healthy subjects, which may be specific to the posterior language region. Another potential variation among these studies is the cognitive condition, including the resting-state (DMN) condition. During the performance of attention-demanding cognitive tasks, specific brain regions exhibit an increase in activity (task-positive regions), whereas other regions exhibit task-related decreases in activity (task-negative regions). The task-negative network that has been implicated in self-referential mental activity includes the DMN, which exhibits increased activity at rest compared with during the performance of various goal-directed tasks (Gusnard and Raichle 2001; Raichle et al. 2001). Human cognitive functions responsible for behavioral control result from the dynamic interplay of distinct cortical systems; for example, a goal-directed (task-positive) network and the DMN or a resting-state (task-negative) network representing opposing components of human mental activity. Successful task performance depends on engaging task-positive network activity while simultaneously suppressing task-negative network activity (Fox et al. 2005).

A study by Tunbridge et al. (2013) indicated that *COMT* Val^158^Met-associated differences were present in the functional connectivity of the PFC even at rest. Furthermore, Meyer et al. (2014) demonstrated that healthy adolescent (14 years) and adult resting-state networks are dose-dependently and diametrically affected by the *COMT* genotype following a hypothetical model of dopamine function that follows an inverted U-shaped curve. Val homozygous adults exhibited increased connectivity, whereas adolescents exhibited decreased connectivity compared with Met homozygotes. Recent studies have reported a stronger functional coupling for adult Val carriers in regions engaged in cognitive tasks (Sambataro et al. 2009; Lee et al. 2011; Tunbridge et al. 2013). Meyer et al. (2014) have suggested that the increased functional connectivity identified in their study and previous studies may be related to the reports of Val allele-dependent increases in cognitive task activation in the PFC, which have been interpreted as “inefficient” PFC functions and thus likely reflect suboptimal dopamine signaling (Egan et al. 2001; Sambataro et al. 2009).

In the present study, the significantly decreased activation in Val homozygous children relative to Met carriers appears to be consistent with Val homozygous adolescents at age 14 who exhibited decreased connectivity associated with decreased task-related activation (efficient functioning) compared with Met carriers. In addition, recent studies suggest that dopamine plays a key role in switching or coordinating the transition between 2 states: resting (task-negative) and task-relevant (task-positive) states (Cole et al. 2011; Dang et al. 2012). Based on these findings, less functional connectivity in the Val allele among preadolescents and adolescents may reflect less functional connectivity between task-positive and task-negative regions. Thus, near optimal coupling at rest in Val homozygotes may beneficially affect the brain's ability to uncouple task-positive and task-negative regions to perform a specific task, whereas superoptimal coupling at rest in Met carriers may detrimentally affect the brain's ability to uncouple task-positive and task-negative regions during task performance. Therefore, the increased activation in the posterior cortical regions in Met carriers relative to Val homozygotes demonstrated in the present study may be a result of increased resting-state connectivity and switching failure between task-negative and task-positive states or a failure to suppress the DMN because of excessive dopamine.

### Strengths and Limitations of the Study

Few genetic studies have examined language development in children with a relatively large sample of brain-based intermediate phenotype data. In general, functional neuroimaging studies of children using fMRI, positron emission tomography, and other approaches pose technical challenges because the head position must be strictly fixed and vocalization may induce severe motion artifacts. Thus, the cognitive tasks used during the measurement of brain functions are limited, and data affected by motion artifacts cannot be used, which makes the sample size small. By contrast, fNIRS is completely noninvasive, and a participant's motion during measurement is tolerated to a higher degree. This approach enabled us to achieve long-duration, real-time monitoring of brain hemodynamics of active and restless healthy young children using a language task with articulation and obtain a relatively large sample of cortical language function. However, the current study has several limitations: 1) fNIRS cannot measure responses in deep brain or subcortical structures. Therefore, the effects of the *COMT* genotype that may exist in subcortical regions could not be detected. 2) The present study demonstrated the effects of the *COMT* genotype on cortical responses during word processing. However, language is complex and consists of more than only a collection of disconnected words; it also consists of different systems, such as phonology, semantics, and syntax. Further studies are necessary to clarify the effects of the *COMT* genotype on both discrete and integrated components of language. 3) The present study demonstrated age-dependent *COMT* genotype effects on language functions. However, the age range investigated was very narrow. Future studies using a wider age range of children will identify a more detailed description of the effects. 4) Only a single *COMT* SNP (rs4680: Val^158^Met) was evaluated in this study. In the *COMT* gene, the Val^158^Met is the most investigated genetic variant in terms of verbal/language ability, perception, or processing; however, there are other functionally important polymorphisms, including rs4818, that have substantial effects on the free energy of mRNA secondary structures (Nackley et al., 2006). In future studies, assessing the effects of these SNPs would be beneficial. 5) The present results were discussed by considering dopaminergic neuronal modulation; however, the *COMT* polymorphism may also affect cortical activity in the posterior language regions by influencing the metabolism of norepinephrine (Mannisto and Kaakkola 1999; Tunbridge et al. 2006) because dense norepinephrine inputs are located in the temporal–parietal region. Further studies are necessary to unravel the precise mechanisms.

## Conclusions

The present study demonstrated that *COMT* influences language performance and cortical responses during language processing that engages posterior language regions in children. Importantly, the *COMT* genotype effects on language performance and cortical responses were found to vary even within a narrow age window of 6–10 years. The slower language development in Val homozygotes compared with Met carriers in the early elementary school years appeared to be due to decreased dopaminergic neurotransmission. In contrast, an increase in D1 receptor density around preadolescence would be of particular relevance to the genotype by age interactions on language performance and processing around this age. Although previous studies have provided evidence for the *COMT* genotype effects on prefrontally mediated cognitive functions, the impact of the *COMT* genotype on cognition was not limited to the PFC but extended to the posterior cortical regions. The present study also suggests the possibility of direct or indirect modulation of posterior activation without innervation of the PFC and the interplay of task-relevant (task-positive) and task-irrelevant (task-negative) activation mediated by DMN.

## Supplementary Material

Supplementary Data

Supplementary Data

Supplementary Data

Supplementary Data

Supplementary Data
