# Supplementary material for: Age-Dependent Effects of Catechol-O-Methyltransferase (COMT) Gene Val158Met Polymorphism on Language Function in Developing Children
Source: Cereb Cortex. 2016 Nov 30;27(1):104–16. doi: 10.1093/cercor/bhw371 (PMC6044402; doi:10.1093/cercor/bhw371)
Supplement: Supplementary Data [file supplementaryfig1_figurelegend.docx]

**Supplementary Figure 1. Semantic knowledge of high-frequency and low-frequency words.**

The percentage of words that the children could define was measured in each condition. The mean semantic knowledge was significantly increased for the high-frequency words compared with the low-frequency words (*P* < 0.001). Error bars indicate the standard deviation.
