# Supplementary figures and images for: Age-Dependent Effects of Catechol-O-Methyltransferase (COMT) Gene Val158Met Polymorphism on Language Function in Developing Children
Source: Cereb Cortex. 2016 Nov 30;27(1):104–16. doi: 10.1093/cercor/bhw371 (PMC6044402; doi:10.1093/cercor/bhw371)

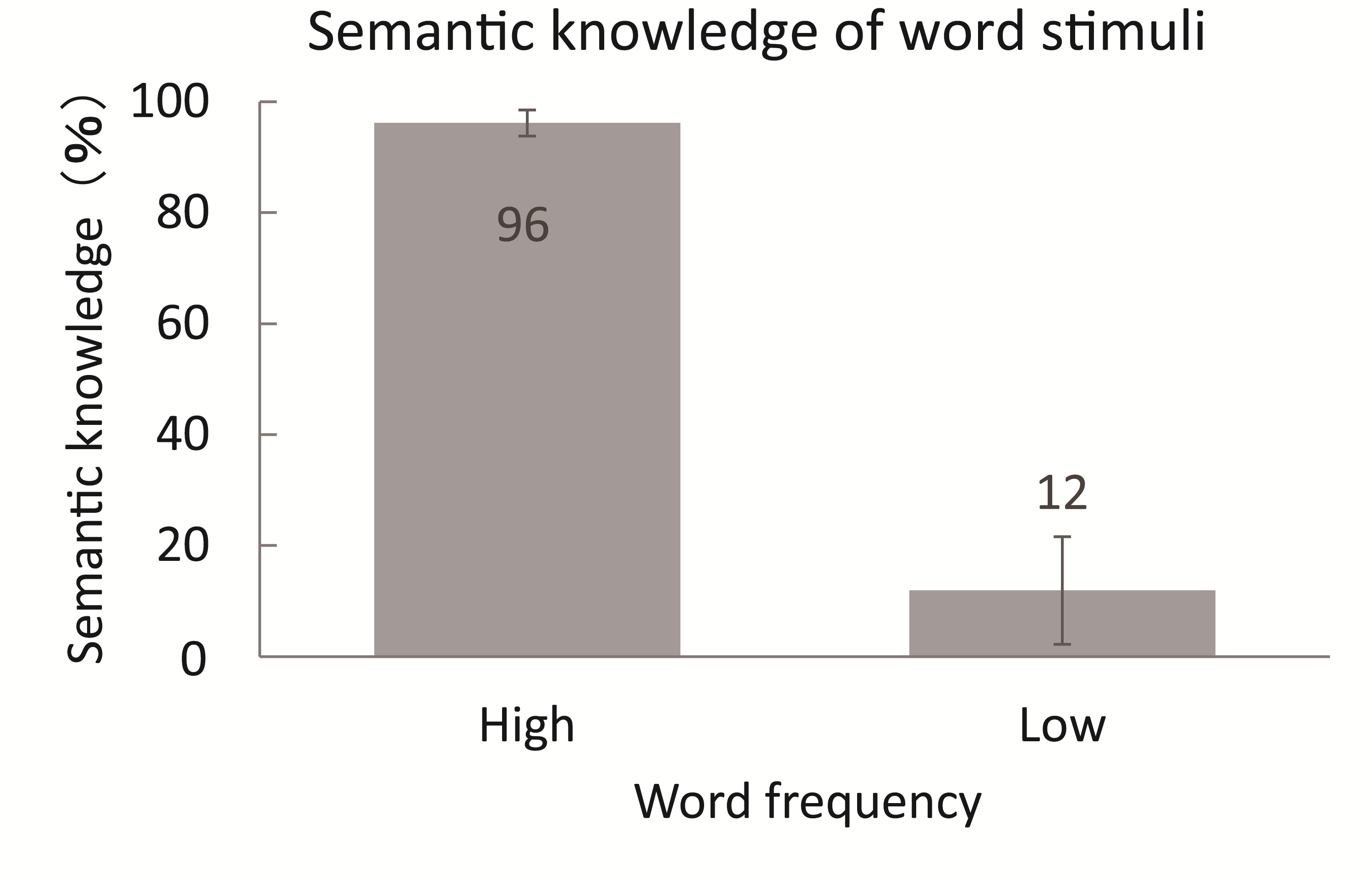

Supplement: Supplementary Data [file supplementaryfig1_lzw_compressed.png]
