# Supplementary material for: Age-Dependent Effects of Catechol-O-Methyltransferase (COMT) Gene Val158Met Polymorphism on Language Function in Developing Children
Source: Cereb Cortex. 2016 Nov 30;27(1):104–16. doi: 10.1093/cercor/bhw371 (PMC6044402; doi:10.1093/cercor/bhw371)
Supplement: Supplementary Data [file supplementarytable1.docx]

**Supplementary Table 1**

**Cortical activation in the independent channels used for the present study**

| Channel No. | High-frequency word condition | | | | | | Low-frequency word condition | | | | | |
| --- | --- | --- | --- | --- | --- | --- | --- | --- | --- | --- | --- | --- |
|  | MM + VM | | | VV | | | MM + VM | | | VV | | |
|  | df | *t* | *P* | df | *t* | *P* | df | *t* | *P* | df | *t* | *P* |
| LH 1 | 127 | 5.597 | < 0.001 | 116 | 3.288 | 0.059 | 127 | 3.879 | 0.007 | 115 | 2.685 | 0.367 |
| LH 3 | 125 | 2.970 | 0.157 | 115 | 1.983 | 2.187 | 127 | .593 | 24.382 | 115 | -.117 | 39.898 |
| LH 4 | 126 | 6.194 | < 0.001 | 116 | 1.497 | 6.031 | 126 | 2.623 | 0.430 | 116 | 2.044 | 1.899 |
| LH 5 | 127 | 2.785 | 0.271 | 114 | 1.431 | 6.834 | 126 | 1.277 | 8.970 | 114 | .640 | 23.021 |
| LH 6 | 123 | 10.268 | < 0.001 | 115 | 7.931 | < 0.001 | 124 | 10.110 | < 0.001 | 114 | 8.322 | < 0.001 |
| LH 8 | 123 | 5.856 | < 0.001 | 115 | 4.830 | < 0.001 | 126 | 3.211 | 0.074 | 116 | 3.363 | 0.046 |
| LH 9 | 126 | 3.651 | 0.017 | 116 | -.453 | 28.676 | 127 | .780 | 19.231 | 116 | .915 | 15.932 |
| LH 10 | 82 | 4.667 | 0.001 | 80 | 4.949 | < 0.001 | 89 | 5.877 | < 0.001 | 87 | 6.459 | < 0.001 |
| LH 12 | 125 | 11.014 | < 0.001 | 113 | 9.160 | < 0.001 | 125 | 9.450 | < 0.001 | 112 | 7.978 | < 0.001 |
| LH 13 | 125 | 7.124 | < 0.001 | 115 | 3.979 | 0.005 | 127 | 5.392 | < 0.001 | 116 | 4.354 | 0.001 |
| LH 14 | 104 | .484 | 27.694 | 100 | 1.329 | 8.226 | 108 | 2.999 | 0.148 | 97 | 1.784 | 3.415 |
| LH 16 | 82 | 8.619 | < 0.001 | 74 | 6.049 | < 0.001 | 86 | 8.964 | < 0.001 | 76 | 7.100 | < 0.001 |
| LH 17 | 124 | 11.048 | < 0.001 | 113 | 7.841 | < 0.001 | 126 | 9.626 | < 0.001 | 112 | 8.985 | < 0.001 |
| LH 21 | 115 | 4.998 | < 0.001 | 108 | 1.536 | 5.605 | 118 | 5.734 | < 0.001 | 103 | 3.274 | 0.064 |
| RH 1 | 125 | 8.611 | < 0.001 | 116 | 8.728 | < 0.001 | 126 | 8.808 | < 0.001 | 116 | 8.603 | < 0.001 |
| RH 3 | 125 | 4.295 | 0.002 | 116 | 2.862 | 0.220 | 126 | 3.125 | 0.097 | 116 | 2.538 | 0.549 |
| RH 4 | 127 | 6.394 | < 0.001 | 114 | 1.913 | 2.562 | 128 | 3.635 | 0.018 | 115 | 2.530 | 0.562 |
| RH 5 | 123 | 4.328 | 0.001 | 116 | 3.931 | 0.006 | 127 | 5.519 | < 0.001 | 114 | 5.176 | < 0.001 |
| RH 6 | 124 | 10.233 | < 0.001 | 113 | 9.052 | < 0.001 | 126 | 7.723 | < 0.001 | 114 | 7.986 | < 0.001 |
| RH 8 | 127 | 5.529 | < 0.001 | 116 | 2.670 | 0.381 | 128 | 3.942 | 0.006 | 116 | 3.306 | 0.055 |
| RH 9 | 126 | 3.260 | 0.063 | 114 | -1.469 | 6.362 | 126 | 1.550 | 5.445 | 112 | .907 | 16.108 |
| RH 10 | 92 | 8.117 | < 0.001 | 88 | 5.867 | < 0.001 | 94 | 6.609 | < 0.001 | 83 | 6.005 | < 0.001 |
| RH 12 | 127 | 11.533 | < 0.001 | 116 | 8.444 | < 0.001 | 126 | 10.708 | < 0.001 | 114 | 8.916 | < 0.001 |
| RH 13 | 125 | 3.901 | 0.007 | 116 | .416 | 29.847 | 126 | 3.070 | 0.115 | 116 | 1.844 | 2.978 |
| RH 14 | 99 | 1.640 | 4.586 | 96 | 2.960 | 0.170 | 101 | 2.743 | 0.317 | 95 | 3.589 | 0.023 |
| RH 16 | 90 | 11.654 | < 0.001 | 78 | 7.382 | < 0.001 | 93 | 11.280 | < 0.001 | 84 | 8.240 | < 0.001 |
| RH 17 | 124 | 9.311 | < 0.001 | 113 | 5.306 | < 0.001 | 124 | 8.706 | < 0.001 | 114 | 6.397 | < 0.001 |
| RH 21 | 121 | 6.929 | < 0.001 | 107 | 3.487 | 0.031 | 123 | 7.123 | < 0.001 | 106 | 5.605 | < 0.001 |

Note: The activity recorded during the stimulus and recovery periods was compared with the activity recorded during the baseline periods using Student’s *t*-tests (Bonferroni corrected for 44 tests for 22 x 2 channels in the left and right hemispheres) with a significance level of *P* < 0.05 after multiple comparison correction. Four language-related ROIs (TR, AG, SMG, and FR in Figure 3) were selected bilaterally, referring to an MNI-compatible macroanatomical atlas (Automatic Anatomical Label), in the channels that showed a statistically significant increase in [oxy-Hb] for at least one combination of *COMT* genotype (Met carriers (MM + VM) or Val homozygotes (VV)), word-frequency condition (high-frequency or low-frequency words) and cerebral hemisphere (LH or RH). The channels 12, 16, 17, and 21, the channels 4, 9, and 13, the channels 3 and 8, and the channels 1, 5, 6, 10, and 14 were used for the TR, AG, SMG, and FR, respectively.

LH = left hemisphere, RH = right hemisphere, MM = Met/Met, VM = Val/Met, VV = Val/Val, df = degrees of freedom, *P* = Bonferroni corrected *P* values, TR = temporal region, including Wernicke’s area, AG = angular gyrus, SMG = supramarginal gyrus, and FR = frontal region, including Broca’s area.
