# Supplementary material for: Age-Dependent Effects of Catechol-O-Methyltransferase (COMT) Gene Val158Met Polymorphism on Language Function in Developing Children
Source: Cereb Cortex. 2016 Nov 30;27(1):104–16. doi: 10.1093/cercor/bhw371 (PMC6044402; doi:10.1093/cercor/bhw371)
Supplement: Supplementary Data [file supplementarytable2.docx]

**Supplementary Table 2**

**Results of 4-way repeated-measures ANOVAs, with sex as a covariate, indicate the main effects of the *COMT* genotype on cortical activation during word processing**

| Brain area | Source of variation | | SS | df | MS | F | *P* uncorrected | *P* corrected | Remarks |
| --- | --- | --- | --- | --- | --- | --- | --- | --- | --- |
| TR | Between subjects | COMT | 0.028 | 1, 237 | 0.028 | 7.234 | 0.008 | 0.031* | MM+VM > VV |
|  |  | age | 0.008 | 1, 237 | 0.008 | 2.103 | 0.148 | n.s. |  |
|  |  | COMT * age | 0.002 | 1, 237 | 0.002 | 0.388 | 0.534 | n.s. |  |
|  |  | Sex | 0.004 | 1, 237 | 0.004 | 0.979 | 0.324 | n.s. |  |
|  | Within subjects | task | 0.005 | 1, 237 | 0.005 | 2.846 | 0.093 | n.s. |  |
|  |  | task * sex | 0.010 | 1, 237 | 0.010 | 5.884 | 0.016 | n.s. |  |
|  |  | task * COMT | 0.004 | 1, 237 | 0.004 | 2.149 | 0.144 | n.s. |  |
|  |  | task * age | 0.009 | 1, 237 | 0.009 | 5.559 | 0.019 | n.s. |  |
|  |  | task * COM * age | 0.004 | 1, 237 | 0.004 | 2.140 | 0.145 | n.s. |  |
|  |  | hemisphere | 0.001 | 1, 237 | 0.001 | 0.840 | 0.360 | n.s. |  |
|  |  | hemisphere * sex | 0.001 | 1, 237 | 0.001 | 0.842 | 0.360 | n.s. |  |
|  |  | hemisphere * COMT | 0.000 | 1, 237 | 0.000 | 0.271 | 0.603 | n.s. |  |
|  |  | hemisphere * age | 0.000 | 1, 237 | 0.000 | 0.163 | 0.687 | n.s. |  |
|  |  | hemisphere * COMT * age | 0.002 | 1, 237 | 0.002 | 1.780 | 0.183 | n.s. |  |
|  |  | task * hemisphere | 0.000 | 1, 237 | 0.000 | 0.083 | 0.773 | n.s. |  |
|  |  | task * hemisphere * sex | 0.000 | 1, 237 | 0.000 | 0.181 | 0.671 | n.s. |  |
|  |  | task * hemisphere * COM | 0.000 | 1, 237 | 0.000 | 0.080 | 0.778 | n.s. |  |
|  |  | task * hemisphere * age | 0.001 | 1, 237 | 0.001 | 1.050 | 0.307 | n.s. |  |
|  |  | task * hemisphere * COMT * age | 0.001 | 1, 237 | 0.001 | 1.935 | 0.166 | n.s. |  |
| AG | Between subjects | COMT | 0.041 | 1, 240 | 6.751 | 6.751 | 0.010 | 0.020* | MM+VM > VV |
|  |  | age | 0.006 | 1, 240 | 0.992 | 0.992 | 0.320 | n.s. |  |
|  |  | COMT * age | 0.011 | 1, 240 | 1.745 | 1.745 | 0.188 | n.s. |  |
|  |  | sex | 0.003 | 1, 240 | 0.542 | 0.542 | 0.463 | n.s. |  |
|  | Within subjects | task | 0.001 | 1, 240 | 0.001 | 0.180 | 0.672 | n.s. |  |
|  |  | task * sex | 0.004 | 1, 240 | 0.004 | 1.305 | 0.254 | n.s. |  |
|  |  | task * COMT | 0.019 | 1, 240 | 0.019 | 6.365 | 0.012 | 0.049* |  |
|  |  | task * age | 0.002 | 1, 240 | 0.002 | 0.751 | 0.387 | n.s. |  |
|  |  | task * COMT * age | 0.000 | 1, 240 | 0.000 | 0.080 | 0.777 | n.s. |  |
|  |  | hemisphere | 0.001 | 1, 240 | 0.001 | 2.048 | 0.154 | n.s. |  |
|  |  | hemisphere * sex | 0.000 | 1, 240 | 0.000 | 0.090 | 0.764 | n.s. |  |
|  |  | hemisphere * COMT | 0.001 | 1, 240 | 0.001 | 0.899 | 0.344 | n.s. |  |
|  |  | hemisphere * age | 0.000 | 1, 240 | 0.000 | 0.013 | 0.911 | n.s. |  |
|  |  | hemisphere * COMT * age | 0.000 | 1, 240 | 0.000 | 0.033 | 0.855 | n.s. |  |
|  |  | task * hemisphere | 0.002 | 1, 240 | 0.002 | 4.469 | 0.036 | n.s. |  |
|  |  | task * hemisphere * sex | 0.001 | 1, 240 | 0.001 | 2.005 | 0.158 | n.s. |  |
|  |  | task * hemisphere * COM | 0.000 | 1, 240 | 0.000 | 0.099 | 0.754 | n.s. |  |
|  |  | task * hemisphere * age | 0.000 | 1, 240 | 0.000 | 0.016 | 0.900 | n.s. |  |
|  |  | task * hemisphere * COMT * age | 0.000 | 1, 240 | 0.000 | 0.005 | 0.945 | n.s. |  |
| SMG | Between subjects | COMT | 0.006 | 1, 236 | 0.921 | 0.921 | 0.338 | n.s. |  |
|  |  | age | 0.007 | 1, 236 | 0.980 | 0.980 | 0.323 | n.s. |  |
|  |  | COMT * age | 0.005 | 1, 236 | 0.677 | 0.677 | 0.411 | n.s. |  |
|  |  | sex | 0.000 | 1, 236 | 0.018 | 0.018 | 0.894 | n.s. |  |
|  | Within subjects | task | 0.001 | 1, 236 | 0.001 | 0.210 | 0.647 | n.s. |  |
|  |  | task * sex | 0.003 | 1, 236 | 0.003 | 0.818 | 0.367 | n.s. |  |
|  |  | task * COMT | 0.007 | 1, 236 | 0.007 | 1.973 | 0.161 | n.s. |  |
|  |  | task * age | 0.005 | 1, 236 | 0.005 | 1.432 | 0.233 | n.s. |  |
|  |  | task * COM * age | 0.003 | 1, 236 | 0.003 | 0.745 | 0.389 | n.s. |  |
|  |  | hemisphere | 0.001 | 1, 236 | 0.001 | 0.623 | 0.431 | n.s. |  |
|  |  | hemisphere * sex | 0.001 | 1, 236 | 0.001 | 1.349 | 0.247 | n.s. |  |
|  |  | hemisphere * COMT | 0.000 | 1, 236 | 0.000 | 0.444 | 0.506 | n.s. |  |
|  |  | hemisphere * age | 0.000 | 1, 236 | 0.000 | 0.226 | 0.635 | n.s. |  |
|  |  | hemisphere * COMT * age | 0.002 | 1, 236 | 0.002 | 1.843 | 0.176 | n.s. |  |
|  |  | task * hemisphere | 0.004 | 1, 236 | 0.004 | 6.615 | 0.011 | 0.043* |  |
|  |  | task * hemisphere * sex | 0.000 | 1, 236 | 0.000 | 0.202 | 0.654 | n.s. |  |
|  |  | task * hemisphere * COM | 0.001 | 1, 236 | 0.001 | 1.258 | 0.263 | n.s. |  |
|  |  | task * hemisphere * age | 0.000 | 1, 236 | 0.000 | 0.042 | 0.838 | n.s. |  |
|  |  | task * hemisphere * COMT * age | 0.001 | 1, 236 | 0.001 | 1.150 | 0.285 | n.s. |  |
| FR | Between subjects | COMT | 0.000 | 1, 239 | 0.004 | 0.004 | 0.952 | n.s. |  |
|  |  | age | 0.000 | 1, 239 | 0.090 | 0.090 | 0.764 | n.s. |  |
|  |  | COMT * age | 0.000 | 1, 239 | 0.086 | 0.086 | 0.769 | n.s. |  |
|  |  | sex | 0.001 | 1, 239 | 0.353 | 0.353 | 0.553 | n.s. |  |
|  | Within subjects | task | 0.002 | 1, 239 | 0.002 | 1.593 | 0.208 | n.s. |  |
|  |  | task * sex | 0.007 | 1, 239 | 0.007 | 4.413 | 0.037 | n.s. |  |
|  |  | task * COMT | 0.002 | 1, 239 | 0.002 | 1.634 | 0.202 | n.s. |  |
|  |  | task * age | 0.003 | 1, 239 | 0.003 | 1.809 | 0.180 | n.s. |  |
|  |  | task * COM * age | 0.004 | 1, 239 | 0.004 | 2.561 | 0.111 | n.s. |  |
|  |  | hemisphere | 0.009 | 1, 239 | 0.009 | 13.639 | 0.000 | < 0.001*** | RH > LH |
|  |  | hemisphere * sex | 0.001 | 1, 239 | 0.001 | 0.997 | 0.319 | n.s. |  |
|  |  | hemisphere * COMT | 0.003 | 1, 239 | 0.003 | 5.345 | 0.022 | n.s. |  |
|  |  | hemisphere * age | 0.002 | 1, 239 | 0.002 | 3.069 | 0.081 | n.s. |  |
|  |  | hemisphere * COMT * age | 0.000 | 1, 239 | 0.000 | 0.048 | 0.827 | n.s. |  |
|  |  | task * hemisphere | 0.000 | 1, 239 | 0.000 | 0.269 | 0.605 | n.s. |  |
|  |  | task * hemisphere * sex | 0.000 | 1, 239 | 0.000 | 1.383 | 0.241 | n.s. |  |
|  |  | task * hemisphere * COM | 0.000 | 1, 239 | 0.000 | 0.009 | 0.925 | n.s. |  |
|  |  | task * hemisphere * age | 0.000 | 1, 239 | 0.000 | 0.707 | 0.401 | n.s. |  |
|  |  | task * hemisphere * COMT * age | 0.001 | 1, 239 | 0.001 | 3.326 | 0.069 | n.s. |  |

Note: Statistical analyses using 4-way repeated-measures ANOVAs were conducted for 4 ROIs, with sex as a covariate, to determine the effects of the *COMT* genotype (Met carriers (MM + VM) and Val homozygotes (VV)), age group (young and old), task condition (high-frequency and low-frequency word conditions), and hemisphere (left hemisphere: LH and right hemisphere: RH). *P* values are based on FDR corrections for 4 tests (for 4 ROIs) with a significance level of *P* < 0.05 after multiple comparison correction. Asterisks indicate significant results (**P* < 0.05, ****P* < 0.001), and n.s. indicates not significant. SS = sum of squares, df = degrees of freedom, MS = mean squares, F = variance ratio, TR = temporal region, including Wernicke’s area, AG = angular gyrus, SMG = supramarginal gyrus, and FR = frontal region, including Broca’s area.
