# Supplementary material for: Age-Dependent Effects of Catechol-O-Methyltransferase (COMT) Gene Val158Met Polymorphism on Language Function in Developing Children
Source: Cereb Cortex. 2016 Nov 30;27(1):104–16. doi: 10.1093/cercor/bhw371 (PMC6044402; doi:10.1093/cercor/bhw371)
Supplement: Supplementary Data [file supplementarytable3.docx]

**Supplementary Table 3**

**Repetition success rate (%)**

| Age group | Word frequency | Met/Met + Val/Met | Val/Val | df | *t* | *P* |
| --- | --- | --- | --- | --- | --- | --- |
| Young | High | 99.8 | 99.9 | ---------------------------- | | |
|  | Low | 97.7 | 97.8 | 121 | -0.412 | 0.681 |
| Old | High | 99.8 | 100.0 | ---------------------------- | | |
|  | Low | 98.4 | 98.4 | 121 | -0.047 | 0.963 |

In the high-frequency word condition, the both age groups showed ceiling effects.
